# cGMP-dependent pathway and a GPCR kinase are required for photoresponse in the nematode *Pristionchus pacificus*

**DOI:** 10.1371/journal.pgen.1011320

**Published:** 2024-11-14

**Authors:** Kenichi Nakayama, Hirokuni Hiraga, Aya Manabe, Takahiro Chihara, Misako Okumura

**Affiliations:** 1 Program of Biomedical Science, Graduate School of Integrated Sciences for Life, Hiroshima University, Hiroshima, Japan; 2 Program of Basic Biology, Graduate School of Integrated Sciences for Life, Hiroshima University, Hiroshima, Japan; University of California San Diego, UNITED STATES OF AMERICA

## Abstract

Light sensing is a critical function in most organisms and is mediated by photoreceptor proteins and phototransduction. Although most nematodes lack eyes, some species exhibit phototaxis. In the nematode *Caenorhabditis elegans*, the unique photoreceptor protein *Cel*-LITE-1, its downstream G proteins, and cyclic GMP (cGMP)-dependent pathways are required for phototransduction. However, the mechanism of light-sensing in other nematodes remains unknown. To address this question, we used the nematode *Pristionchus pacificus*, which was established as a satellite model organism for comparison with *C*. *elegans*. Similar to *C*. *elegans*, illumination with short-wavelength light induces avoidance behavior in *P*. *pacificus*. Opsin, cryptochrome/photolyase, and *lite-1* were not detected in the *P*. *pacificus* genome using orthology and domain prediction-based analyses. To identify the genes related to phototransduction in *P*. *pacificus*, we conducted forward genetic screening for light-avoidance behavior and isolated five light-unresponsive mutants. Whole-genome sequencing and genetic mapping revealed that the cGMP-dependent pathway and *Ppa-grk-2*, which encodes a G protein-coupled receptor kinase (GRK) are required for light avoidance. Although the cGMP-dependent pathway is conserved in *C*. *elegans* phototransduction, GRK is not necessary for light avoidance in *C*. *elegans*. This suggests similarities and differences in light-sensing mechanisms between the two species. Using a reverse genetic approach, we showed that gamma-aminobutyric acid (GABA) and glutamate were involved in light avoidance. Through reporter analysis and suppression of synapse transmission, we identified candidate photosensory neurons. These findings advance our understanding of the diversity of phototransduction in nematodes even in the absence of eyes.

## Author summary

Nematodes are a highly diverse group of animals found in a wide variety of habitats and sensory systems. In particular, light-induced behavior has been found to differ among species. The photoreceptor protein and its downstream pathways in *Caenorhabditis elegans* have been identified, revealing unique and distinct characteristics compared to those in other animals. However, the mechanisms of photoreception in other nematodes remain largely unknown. This study focused on the analysis of the photoreception mechanisms in *Pristionchus pacificus*, a species for which many genetic and molecular tools are available. Similar to *C*. *elegans*, *P*. *pacificus* also exhibits light avoidance behavior towards short-wavelength light; however, known animal photoreceptor genes could not be identified in the *P*. *pacificus* genome using bioinformatic approaches. Using forward and reverse genetic approaches, we found that certain genes and neurons are required for light avoidance, some of which are conserved in *C*. *elegans* photoreception. These results suggest that the light-sensing mechanisms of *C*. *elegans* and *P*. *pacificus* are similar, yet there are differences between the two species. These findings highlight the various light-sensing mechanisms in nematodes.

## Introduction

Light sensing is important for many animals that use visual information to avoid predators or unfavorable environments, and to find food sources or mating partners. Most animals, including Cnidaria, Ctenophora, and Bilateria, utilize opsins, which belong to the G protein-coupled receptor (GPCR) superfamily, as photoreceptors and downstream signaling pathways. For example, in vertebrate rods and cones, light is absorbed by the retinal chromophore, which binds to opsins. Isomerization of the retinal chromophore causes a conformational change in opsins, which activates downstream signaling pathways such as G proteins and phosphodiesterases (PDEs). This results in a decrease in cyclic GMP (cGMP) levels and the closure of cyclic nucleotide-gated (CNG) channels. Opsins and their downstream signaling pathways have been extensively studied in a wide range of animals, including aspects such as protein structure, signaling mechanisms, and evolution [1,2]. However, opsin-independent phototransduction mechanisms are limited in the animal kingdom, and the details of these mechanisms remain unclear.

Some nematodes have been observed to exhibit photoresponses despite the absence of eyes [3–6]. In particular, the nematode *Caenorhabditis elegans* displays various responses to short-wavelength light, including avoidance behavior [7,8], stopping pharyngeal pumping [9], and spitting out food [10,11]. Furthermore, *C*. *elegans* can discriminate between colors [12,13]. Forward genetic screening using light-avoidance behavior has identified a novel photoreceptor protein, *Cel*-LITE-1 [14,15]. In silico prediction of the protein structure and in vivo ectopic expression analysis suggest that *Cel*-LITE-1 is a member of the 7-transmembrane-domain ion channels (7TMICs) and forms a tetramer [16,17]. Similar to GPCRs, 7TMICs have seven transmembrane domains, but their membrane topology is opposite to that of GPCRs, with the N- and C-termini located intracellularly and extracellularly, respectively [17–21]. Putative binding sites for the chromophore and the aromatic amino acids necessary for light absorption have been identified [16,19]. Although *Cel*-LITE-1 is not predicted to be a GPCR, downstream phototransduction of *Cel*-LITE-1 in *C*. *elegans* ASJ neurons, one of the photosensory cells, requires G proteins and the cGMP-dependent pathway (Fig 3C) [7,15]. It is predicted that *Cel-*LITE-1 transduces light stimuli mediated by G-protein α-subunits (*Cel-*GOA-1 and *Cel-*GPA-3) and guanylate cyclases (*Cel-*DAF-11 and *Cel*-ODR-1), resulting in the production of cGMP. Elevated cGMP levels lead to the opening of CNG channels (*Cel*-TAX-2 and *Cel*-TAX-4), causing an influx of calcium ions into the cell. In contrast to vertebrate rods and cones, PDEs (*Cel*-PDE-1, *Cel*-PDE-2, and *Cel*-PDE-5) are not necessary for light response in *C*. *elegans* [15], suggesting a unique opsin-independent mechanism of phototransduction. However, the mechanism of the light response in other nematode species that lack conventional opsins and LITE-1 remains unknown.

The diplogastrid nematode *Pristionchus pacificus* has been established as a satellite model organism for comparison with *C*. *elegans* [22–24]. Several genetic tools have been developed for *P*. *pacificus*, such as an annotated genome [25,26], forward and reverse genetics [22,27], and synaptic connectome in pharynx and head neurons revealed using electron microscope [28–30], which provide a suitable model to understand its neural response and behavioral evolution [31–40]. Both *C*. *elegans* and *P*. *pacificus* have 12 pairs of amphid neurons, and putative amphid neuronal homologs have been identified between these two species [28]. However, it is likely that the functions of amphid neurons differ between the two species, which is supported by the fact that ciliary terminal structures and the expression of amphid neuron-specific genes vary between the two species [28]. It is currently unknown whether *P*. *pacificus* has the ability to sense and respond to light.

Here, we found that *P*. *pacificus* avoids short-wavelength light, although we did not find a conventional opsin, cryptochrome/photolyase, or *lite-1* in the *P*. *pacificus* genome. Forward genetic screening revealed that the cGMP-dependent pathway and a GPCR kinase (GRK) are necessary for light avoidance. In addition, a reverse genetic approach has shown that the neurotransmitters gamma-aminobutyric acid (GABA) and glutamate play a role in light avoidance. These genes were expressed in five amphid neurons, and the inhibition of neurotransmission in these amphid neurons reduced light avoidance.

## Results

### *P*. *pacificus* responds to short-wavelength light

**Fig 1:**
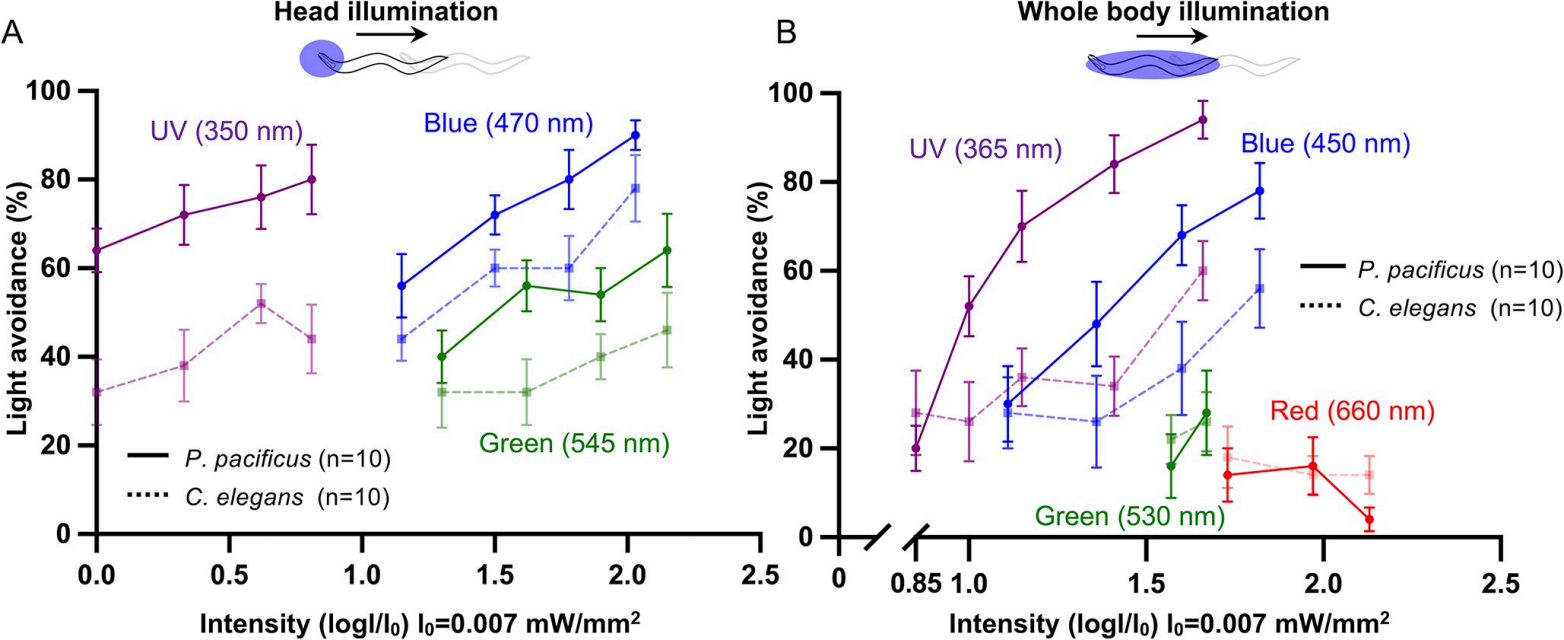
*P*. *pacificus* exhibited avoidance behavior in response to light illumination. (A, B) Results of light avoidance assays using *P*. *pacificus* (straight line) and *C*. *elegans* (dashed lines). UV, blue, green, or red (only B) light illuminated the head (A) or whole body (B) of the worms. *C*. *elegans* and *P*. *pacificus* responded to short wavelength light.

To investigate whether *P*. *pacificus* possesses light-sensing ability, we established a light avoidance assay for *P*. *pacificus* based on previous studies on *C*. *elegans* [7,14]. We illuminated the heads of the worms with different wavelengths of light (UV, blue, and green) for five seconds. In *P*. *pacificus*, exposure of the head of a forward-moving worm to short-wavelength light halted its forward movement and induced backward movement (S1 Movie). The percentage of avoidance increased as the light intensity increased, which is similar to *C*. *elegans* (Fig 1A) [7]. We also illuminated the whole body with different wavelengths of light (UV, blue, green, and red) and observed that *P*. *pacificus* stopped moving forward and began moving backward under UV and blue light illumination (Fig 1B). Avoidance behavior was not induced by illumination of the entire body with green or red light. A previous study reported that whole-body illumination induces forward movement in *C*. *elegans* [14], but in our experiments, *C*. *elegans* moved backward after exposure to light (Fig 1B). For both head and whole-body irradiation, *P*. *pacificus* exhibited a higher percentage of light-avoidance behavior than *C*. *elegans*. (Fig 1A and 1B). These results show that *P*. *pacificus* has the ability to detect light and is more sensitive to light than *C*. *elegans*.

### A combination of orthology and domain prediction could not identify putative photoreceptor proteins in *P*. *pacificus*

**Fig 2:**
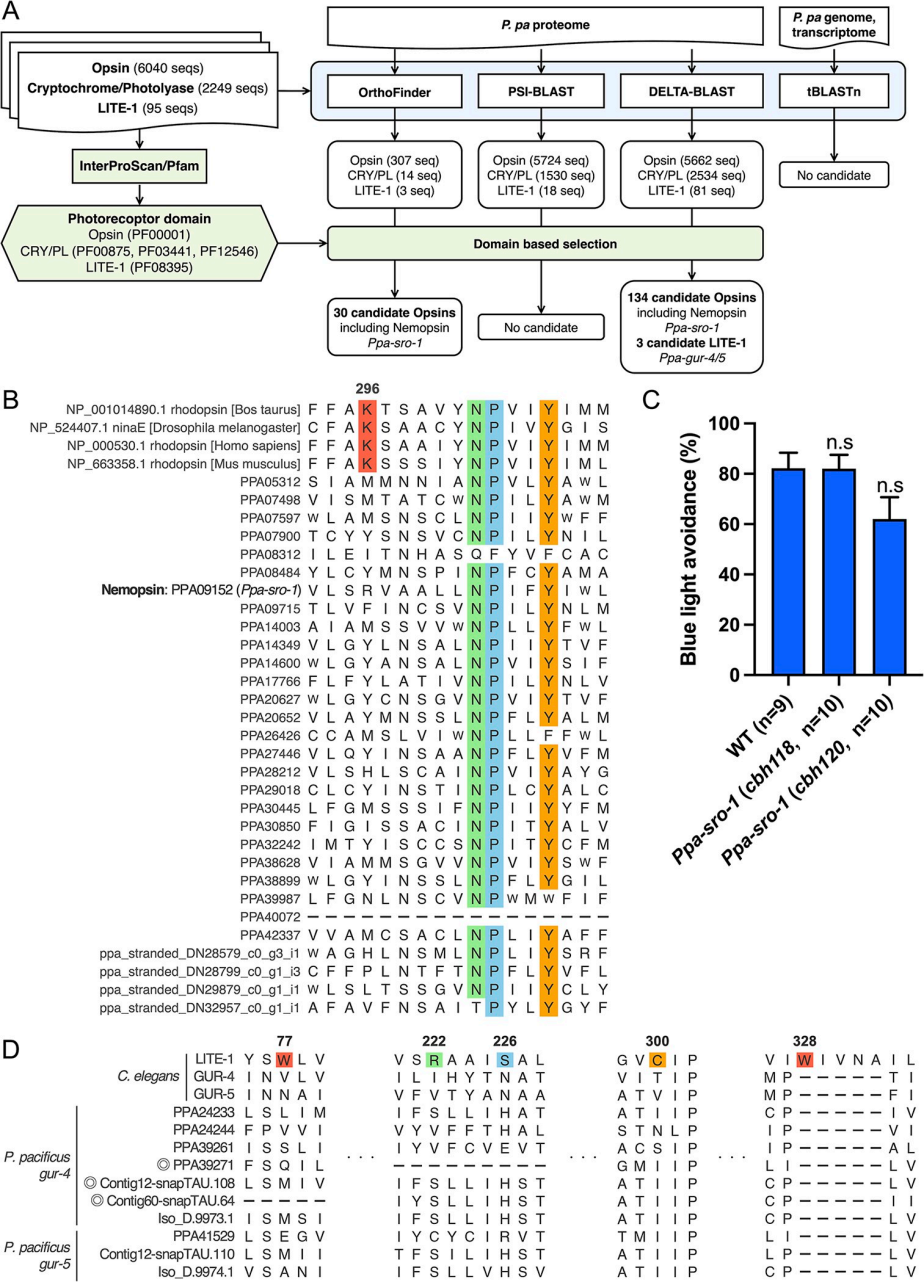
Conserved photoreceptor proteins were not identified in *P*. *pacificus* using a combination of orthology and domain prediction analyses. (A) Search pipeline for known photoreceptors within the *P*. *pacificus* multiome. Using InterProScan/Pfam, protein domains were identified in *P*. *pacificus* proteomes and three photoreceptor families in other animals (Green). CRY/PL; Cryptochrome/Photolyase. Clustering of each photoreceptor family and all *P*. *pacificus* proteins into orthogroups was performed using OrthoFinder (Blue). PSI-/DELTA-BLAST and tBLASTn were performed to search for known photoreceptor proteins in the *P*. *pacificus* multiome (Blue). Finally, data outputs from OrthoFinder and BLAST were utilized to identify *P*. *pacificus* proteins that possess Pfam domains associated with photoreceptors. (B) Sequence alignment of candidate photoreceptor proteins identified by OrthoFinder to the opsin family. While the retinal-binding lysine K296 (*Bos taurus*, red box) is highly conserved among opsins, the candidate GPCRs in *P*. *pacificus* do not have the conserved lysine. The NPxxY motif, which is highly conserved among GPCRs in their seventh transmembrane domain, is colored with green, blue and orange. (C) Blue light avoidance assay for *Ppa-sro-1* mutants. These mutants showed normal percentage of light avoidance. One-way ANOVA, Dunnett’s multiple comparison tests, compared with wild type. n.s. = not significant. (D) Sequence alignment of *Cel-*LITE-1/GUR-4/5 and *Ppa*-GUR-4/5 in the LITE-1 family. Critical amino acid residues (W77, R222, S226, C300, and W328) for photoreception in *Cel-*LITE-1 are not conserved in *Ppa*-GUR-4/5. Circles indicate candidate genes detected in the pipeline.

We investigated whether *P*. *pacificus* possesses any known animal photoreceptor proteins by combining orthology and domain prediction, based on previous studies by Pratx et al, 2018 [41] and Brown et al, 2024 [42] (Fig 2A). We obtained the protein sequences of three animal photoreceptor protein families: opsin, cryptochrome/photolyase, and LITE-1 [19,43]. These sequences and their functional domains were used to search for orthologs in *P*. *pacificus*. Specifically, we evaluated whether each protein in *P*. *pacificu*s: (1) was orthologous to known photoreceptor proteins, and (2) contained protein domains typical of the respective photoreceptor protein families. To conduct these evaluations, we obtained the protein sequences for 6,040 opsins and 2,249 cryptochromes/photolyases from two previously published reference datasets [44,45]. Additionally, 88 sequences representing the LITE-1 family across 28 species were retrieved from WormBase ParaSite [46] using BLASTP with the *Cel-*LITE-1 sequence as the query. This dataset was expanded by adding *Cel-*EGL-47 (also named *gur-1*), a paralog of *Cel-*LITE-1 (*gur-2*), and the sequence of *Gr28b* in *Drosophila melanogaster*, the closest homolog of *Cel-*LITE-1. *Gr28b* has been implicated in UV light sensing in larvae [47]. The final dataset for the LITE-1 family comprised 95 sequences (S1 Data).

We identified the functional protein domains of each photoreceptor family using InterProScan domain prediction and the Pfam database (Fig 2A). Each photoreceptor family had a common functional protein domain: opsins were characterized by the 7-transmembrane receptor (rhodopsin family) (PF00001); cryptochrome/photolyase were identified by three domains—DNA photolyase (PF00875), FAD binding domain of DNA photolyase (PF03441), and Blue/Ultraviolet sensing protein C terminal (PF12546); LITE-1 was defined by the 7tm Chemosensory receptor (PF08395).

We performed a BLASTP search using OrthoFinder, targeting all protein sequences of *P*. *pacificus*, and identified 324 orthogroups. This search yielded 307 hits for opsin, 14 for cryptochrome/photolyase, and three for LITE-1 (S1 Table). Additionally, we conducted both PSI- and DELTA-BLAST searches [48–50] against the *P*. *pacificus* proteome for each known photoreceptor protein because it is possible that *P*. *pacificus* photoreceptor proteins are only distantly related to previously known photoreceptor proteins and might not be detectable by BLASTP alone. PSI-BLAST identified 5,724 hits for opsin, 1,530 for cryptochrome/photolyase, and 18 for LITE-1 (S2 Table). DELTA-BLAST, which uses conserved protein domain databases, detected 5662 hits for opsin, 2534 for cryptochrome/photolyase, and 81 for LITE-1 (S2 Table). Furthermore, to determine whether there were any indications of the loss of known photoreceptor protein genes within the genome, we used tBLASTn to search the photoreceptor protein reference dataset against both the genome (El Paco Assembly) [26] and the transcriptome (El Paco V3) of *P*. *pacificus*. However, the majority of tBLASTn hits overlapped with those identified by PSI-BLAST, and many of the genomic region hits corresponded to either introns or non-coding sequences (S2 Table).

We then checked whether these candidate genes encode the protein domains of characteristic of each photoreceptor protein. Among them, *P*. *pacificus* proteome has 30 genes in OrthoFinder and 134 genes in DELTA-BLAST, respectively, with conserved domains characteristic of opsin family specific domains (PF00001), no genes with the cryptochrome/photolyase, and 3 genes with *lite-1* families (Fig 2A). However, the highly conserved lysine residue among opsins (K296, as found in *Bos taurus*), which enables opsins to bind retinal and is crucial for photoreception [51], was not conserved in these protein sequences (Fig 2B, we showed only 30 sequences identified in OrthoFinder). Among these genes, we identified *Ppa-sro-1*, which belongs to the nemopsin family. Nemopsins are a family of chromopsins that includes peropsins, RGR-opsins, and retinochromes, some of which function as photoreceptor proteins; however, nemopsins feature a substitution of conserved lysine to arginine (Fig 2B) [44]. In *C*. *elegans*, *Cel-sro-1* is expressed in ADL chemosensory neurons and SIA motor neurons [52], but the functions of *Cel-sro-1* and *Ppa-sro-1* are unknown. We performed the blue light avoidance assay with head illumination and the percentage of light avoidance behavior in the *Ppa-sro-1* mutant was similar to that in the wild type (Fig 2C), suggesting that *Ppa-sro-1* is not required for light avoidance in *P*. *pacificus*.

DELTA-BLAST using LITE-1 as a query detected 81 genes (S2 Table), three of which were annotated using protein domain prediction by InterProScan (Fig 2A). To assess their potential as photoreceptors, we compared the crucial amino acid residues for photoreception in *Cel-*LITE-1 (W77, R222, S226, C300, and W328) [16,19] against these three genes and other *Ppa-gur-4/5* homologs (S3 Table) because all three genes are homologous to *Cel-gur-4/5*. However, we found that none of the *Ppa-gur-4/5* genes encode for the amino acid residues essential for photoreception (Fig 2D). These results suggest that, despite using a combined approach of orthology clustering, extensive BLAST searches, domain prediction, and sequence alignment to compare conserved residues, conserved photoreceptor proteins in *P*. *pacificus* could not be identified.

### cGMP-dependent pathway is required for light avoidance in *P*. *pacificus*

**Fig 3:**
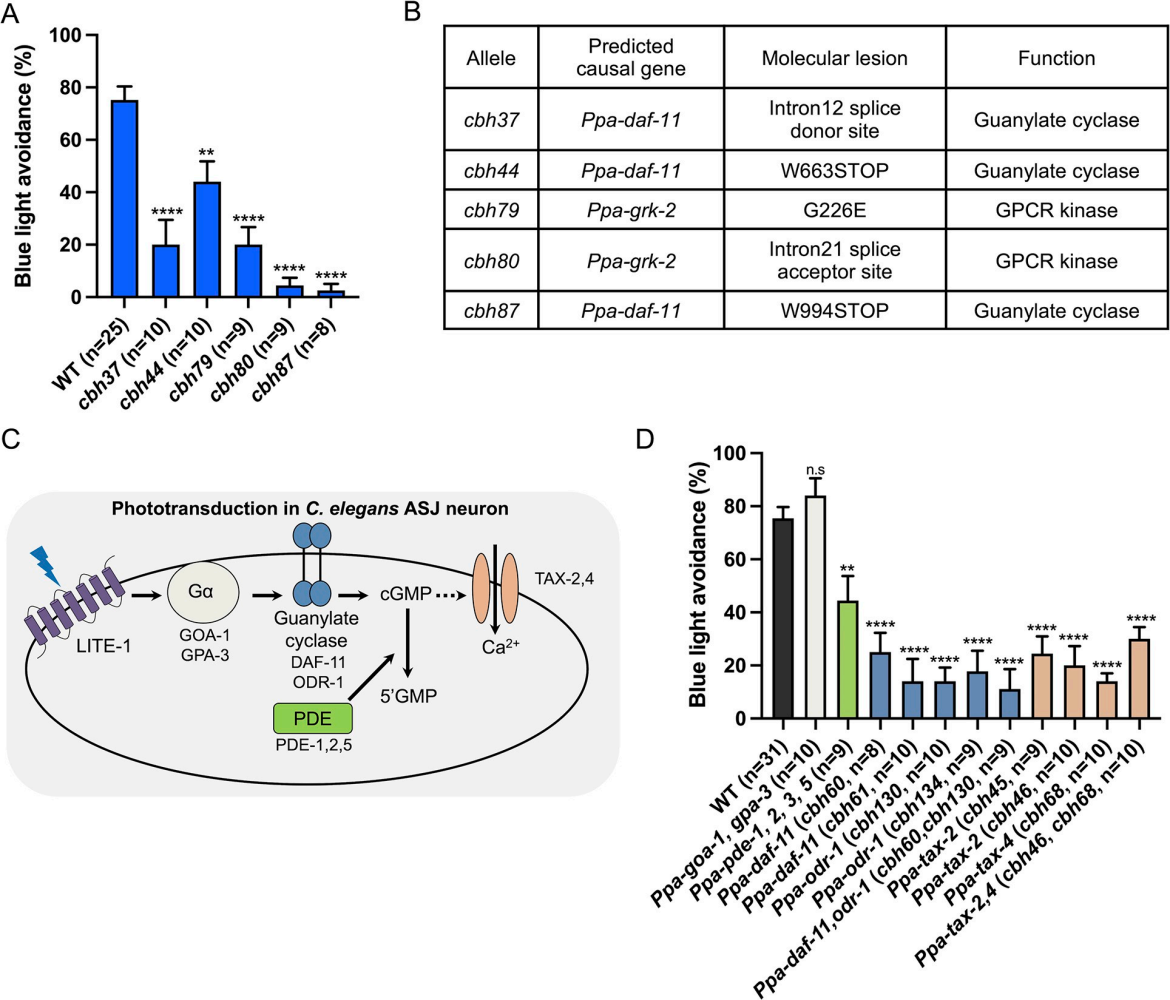
Light avoidance requires the cGMP-dependent pathway in *P*. *pacificus*. (A) Blue light avoidance assay for light-unresponsive mutants that were isolated by forward genetic screening. These mutants had decreased light-induced avoidance response. (B) List of predicted causal genes. (C) Scheme of phototransduction in the *C*. *elegans* ASJ neurons. (D) Blue light avoidance assay for mutants of cGMP-dependent pathway genes. Mutants of guanylate cyclases, CNG channels, and PDEs had defect in light avoidance in *P*. *pacificus*. G-protein α-subunit mutants displayed normal light avoidance. One-way ANOVA, Dunnett’s multiple comparison tests, compared with wild type. n.s = not significant, **P < 0.01, ****P < 0.0001.

To identify the genes responsible for light avoidance in *P*. *pacificus*, we conducted forward genetic screening using light avoidance behavior. We mutagenized wild-type *P*. *pacificus* using ethyl methanesulfonate (EMS) and tested the light-avoidance behaviors of F2 and F3 animals. We screened more than 20,000 strains and identified five light-unresponsive mutants. These mutants exhibited a decreased percentage of light avoidance (Fig 3A). Whole-genome sequencing revealed that *cbh37* harbored a mutation in splice donor site and *cbh44* and *cbh87* had nonsense mutations in *Ppa-daf-11*, which encodes guanylate cyclase (Fig 3B). This gene is a one-to-one ortholog of *Cel-daf-11* and is involved in the phototransduction of the ASJ sensory neurons in *C*. *elegans* (Fig 3C) [15]. This led us to hypothesize that *P*. *pacificus* uses a similar phototransduction to *C*. *elegans*. Since the G-protein α-subunit and the cGMP-dependent pathway are used in *C*. *elegans* phototransduction [7,15], we generated knock-out mutants of the G-protein α-subunit (*Ppa-goa-1*; *Ppa-gpa-3* double), guanylate cyclases (*Ppa-odr-1*, *Ppa-daf-11*, and *Ppa-odr-11*; *Ppa-daf-11* double), CNG channels (*Ppa-tax-2*, *Ppa-tax-4*, and *Ppa-tax-2*; *Ppa-tax-4* double), and cGMP specific PDEs (*Ppa-pde-1*; *Ppa-pde-2*; *Ppa-pde-3*; *Ppa-pde-5* quattro) using the CRISPR/Cas9 system (Fig 3C). While G-protein α-subunit mutants displayed normal light avoidance, mutants of guanylate cyclases, CNG channels, and the PDEs decreased the percentage of light avoidance (Fig 3D). These results support our hypothesis that *P*. *pacificus* and *C*. *elegans* exhibit conserved cGMP-dependent phototransduction.

### GPCR kinase *grk-2* is required for light avoidance in *P*. *pacificus* but not in *C*. *elegans*

**Fig 4:**
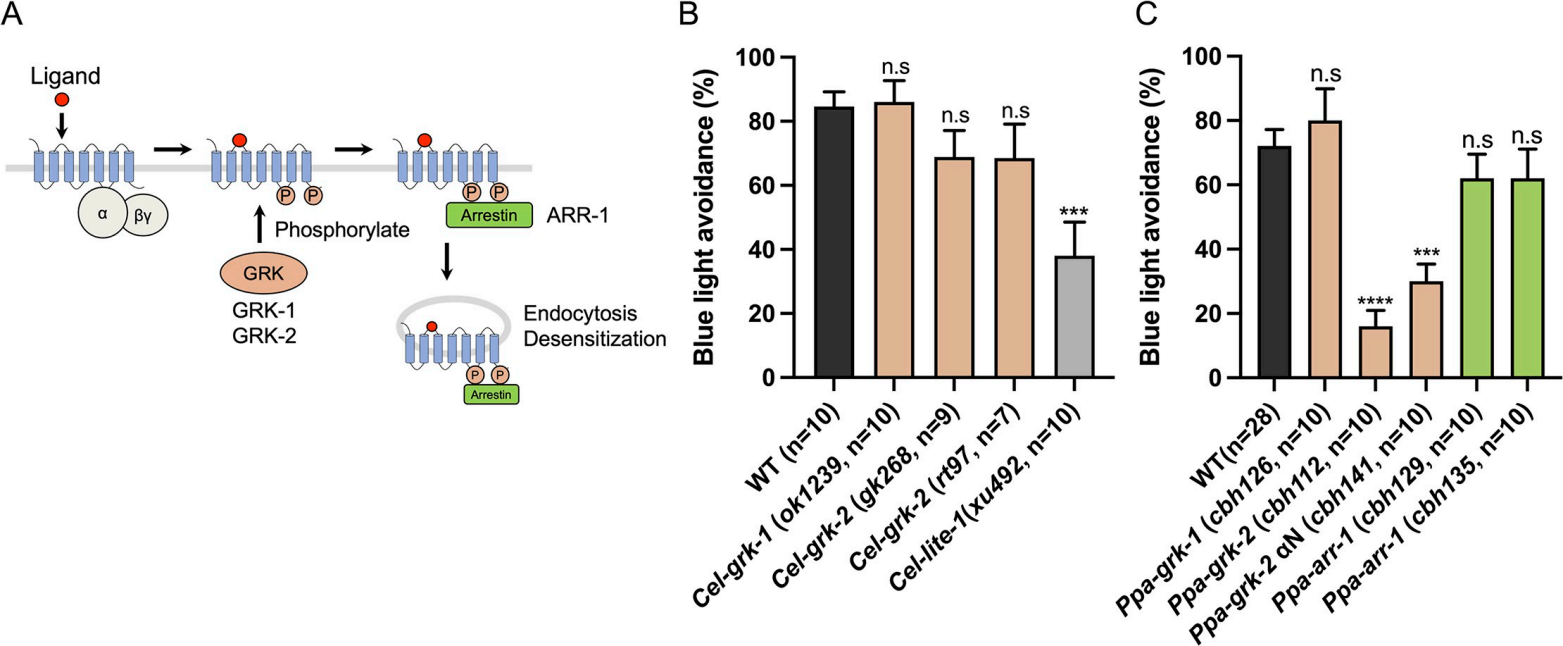
GPCR kinase, *Ppa*-GRK-2 is required for light avoidance. (A) Schematic of GPCR desensitization by GRKs. After a ligand binds to a GPCR, G proteins are released from the activated GPCR to transmit signaling. GRKs phosphorylate the activated GPCR, promoting the binding of arrestin to the receptor. GPCR is endocytosed and desensitized. (B) Blue light avoidance assay for GRK and arrestin mutants in *C*. *elegans*. These mutants displayed normal light avoidance. The *Cel-lite-1* mutants were used as a negative control. (C) Blue light avoidance assay for GRK and arrestin mutants in *P*. *pacificus*. *Ppa-grk-2* mutants had decreased light avoidance. One-way ANOVA, Dunnett’s multiple comparison tests, compared with wild type. n.s = not significant, ***P < 0.001, ****P < 0.0001.

Genetic mapping revealed that EMS mutants, *cbh79* and *cbh80*, harbored a missense mutation and a splice acceptor site mutation, respectively, in *Ppa-grk-2*, which encodes a G protein-coupled receptor kinase (GRK) (Fig 3B). GRKs play a crucial role in the regulation of GPCRs by phosphorylating activated GPCRs, subsequently leading to the binding of arrestin to GPCR. This interaction induces endocytosis and desensitization (Fig 4A) [53]. In vertebrate rod and cone cells, GRKs phosphorylate light-activated opsins, which are crucial for photoreception [54–58]. Both *P*. *pacificus* and *C*. *elegans* possess two GRK genes (*grk-1* and *grk-2*) and an arrestin gene (*arr-1*). In *C*. *elegans*, *Cel-grk-2* is essential for chemosensation [59,60], but *Cel-grk-1* and *Cel-grk-2* mutants showed normal light avoidance (Fig 4B), which is consistent with the fact that the *C*. *elegans* photoreceptor protein *Cel*-LITE-1 is a member of 7TMICs rather than GPCRs [16,17,21].

We generated knock-out mutants in *Ppa-grk-1*, *Ppa-grk-2*, and *Ppa-arr-1* using the CRISPR/Cas9 system in *P*. *pacificus*. We found that the *Ppa-grk-2* mutants (*cbh112*), but not the *Ppa-grk-1* or *Ppa-arr-1* mutants, exhibited decreased light avoidance (Fig 4C), suggesting different roles for *Ppa*-GRK-1 and *Ppa*-GRK-2. We also generated a *Ppa-grk-2* mutant (*cbh141*) lacking a part of the αN domain that stabilizes its binding to activated GPCRs. The absence of the αN domain resulted in a reduction of light avoidance (Fig 4C), implying that the potential involvement of GPCR phosphorylation in light avoidance mechanism in *P*. *pacificus*. Together, these results indicate that the GRK-2, particularly its αN domain, is necessary for light avoidance in *P*. *pacificus* but not in *C*. *elegans*.

### Neurotransmitters GABA and glutamate are required for light avoidance

**Fig 5:**
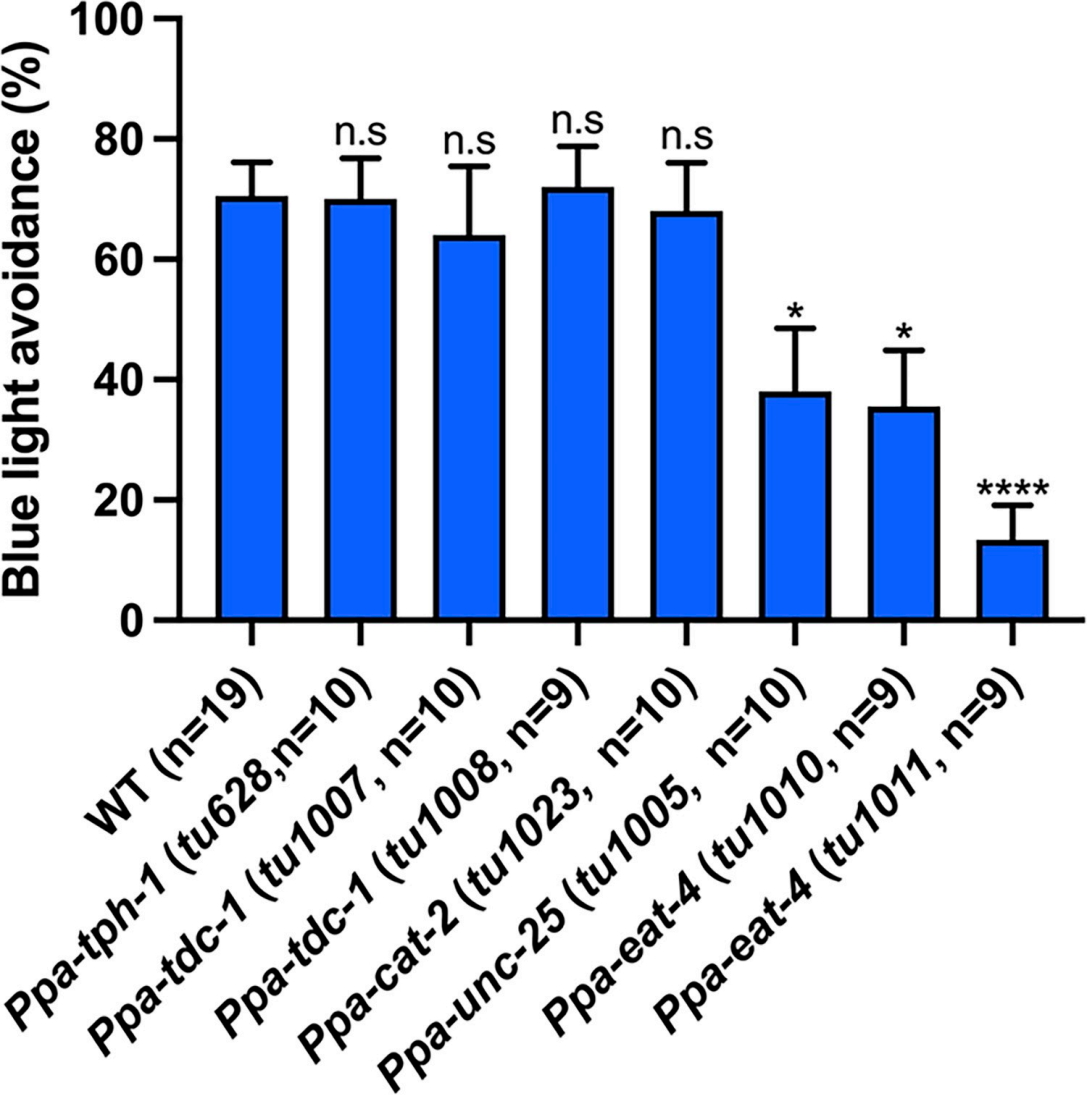
GABA and glutamate are required for light avoidance. Blue light avoidance assay for neurotransmitter-related mutants. *Ppa-unc-25* and *Ppa-eat-4* mutants exhibit decreased light avoidance. One-way ANOVA Dunnett’s multiple comparison tests, compared with wild type. n.s = not significant, *P < 0.05, ****P < 0.0001.

To identify the genes involved in light avoidance, we focused on neurotransmitters. In *C*. *elegans*, neurotransmitters, glutamate, and glutamate receptors are involved in light-avoidance behavior [8]. We performed the light-avoidance assay using mutants of the following neurotransmitter-related genes: *Ppa-tph-1* encoding a tryptophan hydroxylase responsible for serotonin synthesis, *Ppa-tdc-1* encoding a tyrosine decarboxylase required for tyramine and octopamine synthesis, *Ppa-cat-2* encoding a tyrosine hydroxylase required for dopamine synthesis, *Ppa-unc-25* encoding a GABA synthesis enzyme, and *Ppa-eat-4* encoding a vesicular glutamate transporter [31,61]. The mutants *Ppa-tph-1*, *Ppa-tdc-1*, and *Ppa-cat-2* did not show a significant difference in the percentage of light avoidance compared to the wild-type (Fig 5). In contrast, mutants of *Ppa-unc-25* and *Ppa-eat-4* exhibited a decrease in the percentage of light avoidance (Fig 5). These findings indicated that both GABA and glutamate are involved in light avoidance in *P*. *pacificus*.

### Amphid neurons expressing *Ppa-daf-11*, *tax-2*, *tax-4*, and *grk-2* mediate light avoidance behavior

**Fig 6:**
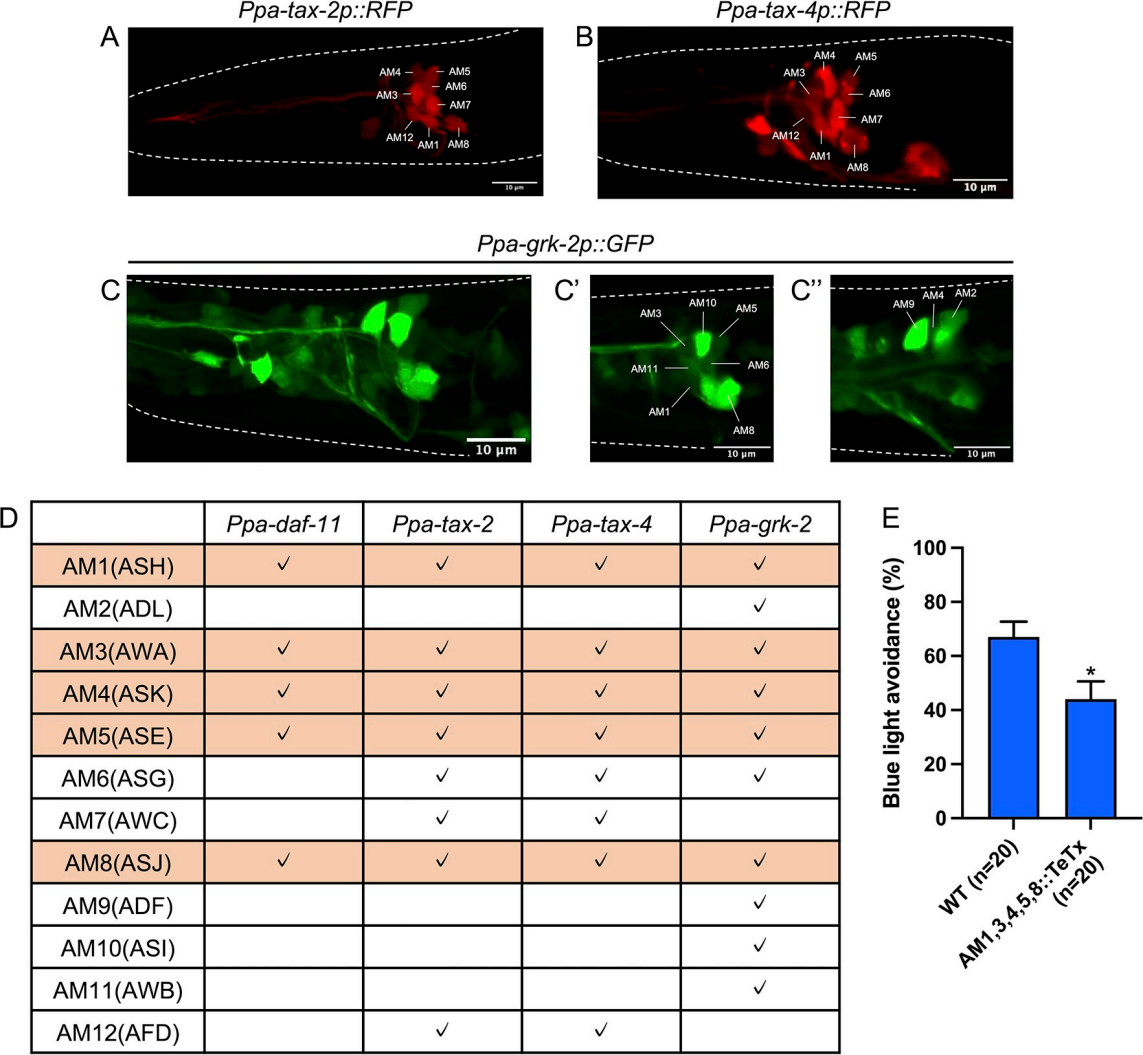
Amphid neurons expressing guanylate cyclase, CNG channels, and GRK-2 are important for light avoidance. (A–C) Representative fluorescence images of *Ppa-tax-2p*::*RFP* (A), *Ppa-tax-4p*::*RFP* (B), and *Ppa-grk-2p*::*GFP* (C) in the head region. Anterior is left and dorsal is up. (A–C) are maximum projection images. (C′) and (C′′) are single focal plane images. (D) Summary of the expression pattern of phototransduction genes. Check marks indicate that the reporter fluorescent proteins were expressed in the corresponding cells in more than 80% individuals. All four phototransduction genes we examined were expressed in AM1, 3, 4, 5, and 8 neurons (orange rows). The expression of *Ppa-daf-11* was examined in a previous study [62]. *Ppa-tax-2p*::*RFP*; n = 17 (*excbh23*), n = 20 (*excbh24*), *Ppa-tax-4p*::*RFP*; n = 18, *Ppa-grk-2p*::*GFP*; n = 19. (E) Blue light avoidance assay for worms expressing tetanus toxin in AM1, 3, 4, 5, and 8 neurons using the *Ppa-daf-11* promotor. The transgenic animals had decreased light avoidance. Student’s *t*-test. *P < 0.05.

To identify the photosensory neurons, we generated transgenic reporter lines for *Ppa-tax-2*, *Ppa-tax-4*, and *Ppa-grk-2*. We found that *Ppa-tax-2* and *Ppa-tax-4* were expressed in the head and amphid neurons (Figs 6A, 6B, S1A and S1B). *Ppa-grk-2* showed a broader expression pattern, including in amphid neurons, pharyngeal muscles, head neurons, body wall muscles, and ventral nerve codes (Figs 6C and S1C–S1E). Since in *C*. *elegans*, amphid neurons such as ASJ, ASK, and ASH neurons are photosensory neurons [15], we focused on the amphid neurons of *P*. *pacificus* for further cell identification. Previous studies have shown that *Ppa-daf-11* is expressed in AM1, 3, 4, 5, and 8 neurons [62]. Our reporter lines revealed that *Ppa-tax-2* and *Ppa-tax-4* were expressed in AM1, 3, 4, 5, 6, 7, 8, and 12 neurons while *Ppa-grk-2* was expressed in AM1, 2, 3, 4, 5, 6, 8, 9, 10, and 11 neurons (Fig 6D). AM1, 3, 4, 5, and 8 neurons expressed all the four genes (Fig 6D). These results suggest that these amphid neurons are potential photosensory neurons.

Amphid neurons in *P*. *pacificus* are ciliated [28] and are important for detecting environmental stimuli [40,63,64]. To investigate the role of cilia in light detection, we examined light avoidance in cilia-related mutants [40,63]. We used mutants of *Ppa-daf-19*, encoding a regulatory factor X transcriptional factor, and several intraflagellar transport components including IFT-B (*Ppa-osm-1*), BBsome (*Ppa-osm-12*), Kinesin-2 (*Ppa-klp-20*), and Dynein-2 (*Ppa-che-3*). These mutants had a defect in dye filing (S2A–S2F Fig), which is consistent with the previous studies of mutants in cilia-related genes [40,63]. These mutants exhibited normal light avoidance (S2G Fig), showing that cilia are not necessary for light avoidance in *P*. *pacificus*.

To examine whether these amphid neurons are necessary for light avoidance, we used tetanus toxin to inhibit the release of neurotransmitters and neuropeptides in specific cells [65]. We expressed the codon-optimized tetanus toxin in AM1, 3, 4, 5, and 8 neurons by utilizing the *Ppa-daf-11* promoter. This transgenic strain exhibited reduced light-avoidance (Fig 6E). Taken together, these results suggest that these cells are candidate photosensory neurons that mediate light avoidance in *P*. *pacificus*.

## Discussion

**Fig 7:**
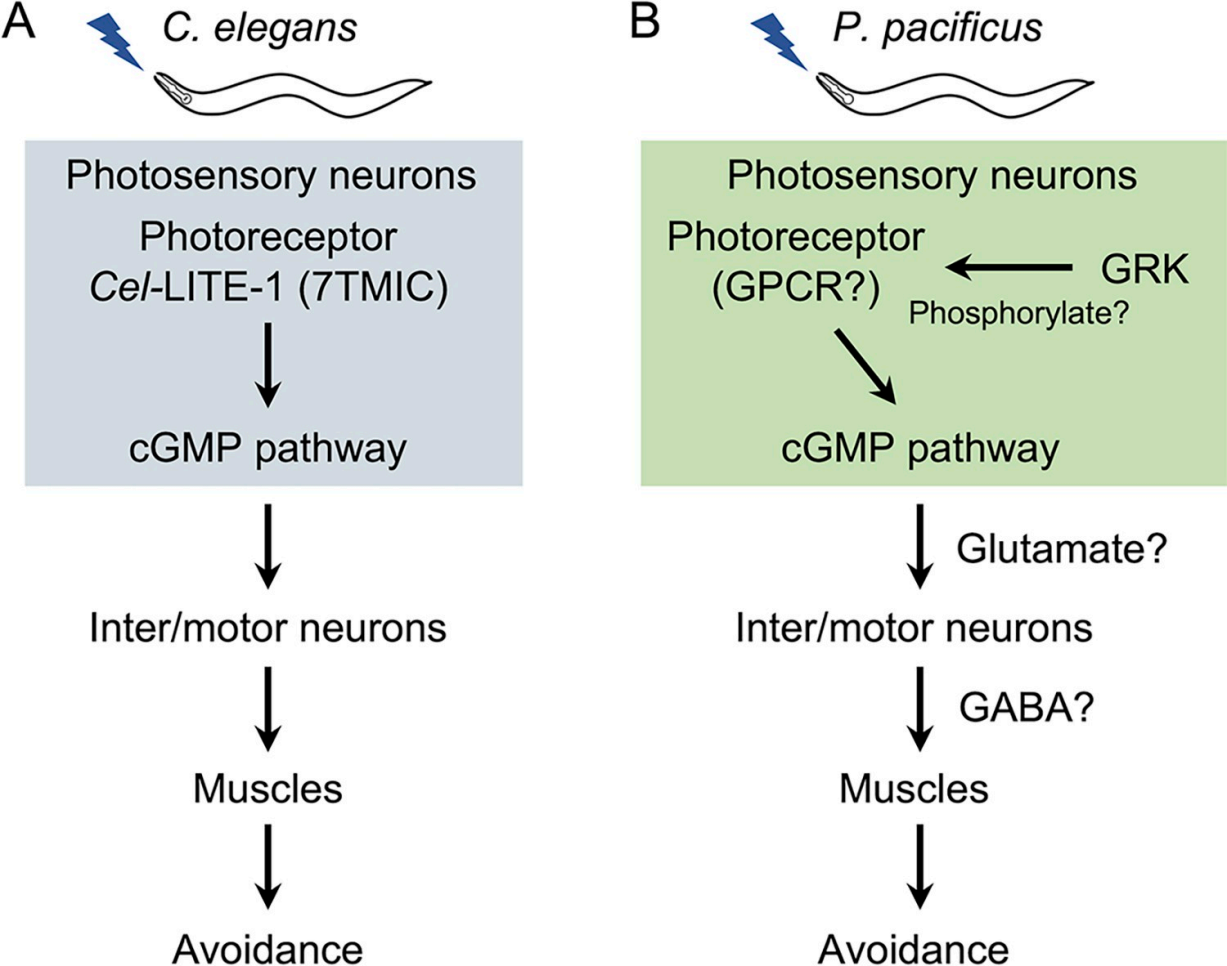
Proposed regulatory models of light avoidance behavior in *C*. *elegans* and *P*. *pacificus*. (A) In *C*. *elegans*, *Cel*-LITE-1, a photoreceptor protein in photosensory neurons (ASJ and ASK) receives light and transmits the signal to the downstream cGMP-dependent pathway. Signals from the photosensory neurons are transmitted to the interneurons and motor neurons. (B) In *P*. *pacificus*, unknown photoreceptor proteins in the photosensory neurons (AM1, 3, 4, 5, or 8) detect light and transmit the signal to the downstream cGMP-dependent pathway. Signals from photosensory neurons are transmitted to interneurons and motor neurons via glutamate or GABA, leading to the muscle contraction and avoidance behavior. GRKs may phosphorylate photoreceptor proteins.

*C*. *elegans* utilizes a unique *Cel-*LITE-1-dependent photoreception instead of conventional animal photoreceptor proteins such as opsin and cryptochrome/photolyase. However, it remains unclear how other nematodes sense light. In the present study, we identified the genes and neurons that mediate light-avoidance behavior in the diplogastrid nematode *P*. *pacificus*. Although the combination of orthology and domain prediction could not identify opsin, cryptochrome/photolyase, or *lite-1* in its genome (Fig 2), *P*. *pacificus* responds to light, suggesting that *P*. *pacificus* possesses photoreceptor proteins that differ from known photoreceptor proteins. Using forward and reverse genetic approaches, we found that the cGMP-dependent pathway, *Ppa-*GRK-2, GABA, glutamate, and some amphid neurons mediate light-avoidance behavior in *P*. *pacificus*. Because glutamate, the cGMP-dependent pathway, and amphid neurons, but not GRKs, are also used for photoresponses in *C*. *elegans* (Fig 4B) [7,8,15], *C*. *elegans* and *P*. *pacificus* have similar but different light-response mechanisms (Fig 7).

The phototransduction of *P*. *pacificus* is similar in some aspects to the previously known phototransduction in *C*. *elegans*. Although *Cel*-LITE-1 has been proposed to function as a light-activated ion channel [16], the G-protein α-subunit and the cGMP-dependent pathway is required for phototransduction downstream of *Cel*-LITE-1 in the ASJ and ASK neurons [7,15]. In *C*. *elegans*, phototransduction does not require PDEs in ASJ neurons [15]. In *P*. *pacificus*, the guanylate cyclases (*Ppa-odr-1*, *Ppa-daf-11*) and CNG channels (*Ppa-tax-2*, *4*) are required for light avoidance behavior (Fig 3D), and quattro mutants of *Ppa-pde-1*, *2*, *3*, and *5* showed a reduced percentage of light avoidance (Fig 3D). These results suggest that, although they share the same cGMP signaling pathway, there may be differences in the activation mechanism of the cGMP signaling pathway.

The cGMP-dependent pathway is crucial for opsin-dependent phototransduction in various animals, including vertebrate rods and cones, marine mollusks, scallops, and lizard parietal eyes [43]. In vertebrate rod and cone cells, GPCR opsins are phosphorylated and desensitized by GRKs [54–58]. The percentage of light avoidance decreased in the *Ppa-grk-2* knock-out mutants and mutants lacking a part of the αN domain, which is thought to be important for GPCR binding (Fig 4C). These results suggest that the photoreceptor protein in *P*. *pacificus* is a GPCR that is phosphorylated by *Ppa*-GRK-2. Nemopsin, encoded by *Ppa-sro-1*, is a recently described opsin that lacks a conserved lysine [44]. Nemopsin has the closest amino acid sequence to opsin among all the GPCRs of *P*. *pacificus*. However, our mutant analysis revealed that *Ppa-sro-1* mutants exhibited normal light avoidance (Fig 2C). It is possible that nemopsin functions redundantly with other photoreceptor proteins, but it is likely that at least one protein that differ from known photoreceptors functions as a photoreceptor in *P*. *pacificus*. Notably, the amino acid sequence of *Cel*-LITE-1, which belongs to the 7TMIC superfamily, differs greatly from that of existing photoreceptor proteins [16,17,21]. Recently, 7TMICs, which were thought to be conserved only in invertebrates, were revealed to be ancient, conserved proteins with highly divergent amino acid sequences by the identification of homologous proteins based on protein structure [17,21]. Future structure-based analyses may allow the identification of photoreceptor proteins that cannot be identified by amino acid sequence homology.

We found that both *Ppa-unc-25*, encoding a synthetic enzyme of the neurotransmitter GABA, and *Ppa-eat-4*, encoding a vesicular glutamate transporter, are required for light avoidance in *P*. *pacificus* (Fig 5). In *C*. *elegans*, *Cel-unc-25* mutants exhibit hypercontraction of body muscles, excessive head bending during foraging, and a significant decrease in contraction of enteric muscles [66]. In addition, the body bending rate was reduced in the *Ppa-unc-25* mutant in *P*. *pacificus* [61] and the expression of *Ppa-unc-25* was observed in RIS and RIB interneurons, RME, DD, and VD motor neurons, and AVL polymodal (motor and inter) neuron [67]. Based on these results, it is likely that the *Ppa-unc-25* mutant is defective in avoidance behavior rather than in the regulation of photoreception. Glutamate and glutamate receptors are required for UV light avoidance behavior in *C*. *elegans* [8]. The photosensory neurons of *C*. *elegans*, specifically the ASK and ASH neurons, are glutamatergic [68]. Additionally, the photoreceptor cells in the mammalian retina are glutamatergic [69]. The body bending rate is normal in *Ppa-eat-4* mutants in *P*. *pacificus* [61], and *Ppa-eat-4* is expressed in head neurons [67]. Although we could not rule out the possibility that the light avoidance defect of *Ppa-eat-4* is due to the activity of an interneuron, it is likely that the photosensory neurons in *P*. *pacificus* are glutamatergic.

We identified candidates for the five photosensory neurons, AM1, 3, 4, 5 and 8. These neurons correspond to the ASH, AWA, ASK, ASE, and ASJ neurons in *C*. *elegans* [28]. The ASK and ASJ neurons are photosensory neurons that utilize the cGMP-dependent pathway for phototransduction [7,15]. Therefore, the corresponding AM4 and 8 neurons are potential candidates as photosensory neurons. In the future, photosensory neurons could be identified through calcium imaging or electrophysiological recordings.

Orthology and domain analyses did not identify a photoreceptor protein in *P*. *pacificus* (Fig 2). This suggested that *P*. *pacificus* has a novel photoreceptor protein. Furthermore, the zoonotic nematode *Dirofilaria immitis* displays positive phototaxis towards infrared light [4]. Although *Cel-*LITE-1 detects UV and blue light, it does not play a role in the perception of longer light wavelengths [16,19]. Therefore, *D*. *immitis* might possess novel photoreceptor proteins. Thus, nematodes possess various photosensory mechanisms and can serve as valuable models for studying the evolution of photoreception.

## Methods

### Strains

The strains used in this study are listed in S4 Table. *C*. *elegans* and *P*. *pacificus* were maintained at 20°C on Nematode Growth Medium (NGM) agar plates with *Escherichia coli* OP50 as previously described [23,70].

### Light avoidance assay

The light avoidance assay was conducted as previously described [7] with some modifications. One-day adult hermaphrodite worms were placed individually on NGM plates covered with a thin bacterial lawn of freshly seeded OP50 and left in the dark for at least 10 min before the assay. For head illumination, in Fig 1A, a fluorescence stereomicroscope (Leica, 165 FC) was connected to a mercury lamp (Leica, EL6000) and the head of the nematode that moved forward was illuminated using a fluorescence filter and an objective lens (Leica, 10450028). Light intensity was adjusted by manipulating the amount of light emitted from the mercury lamp. The following fluorescence filters and wavelengths were used: UV (350 nm), Leica ET UV LP, 1045609; blue (470 nm), Leica ET GFP, 10447408; green (545 nm), Leica ET DSR, 10447412. For other experiments, the LED light source from a fluorescence stereomicroscope (ZEISS, Discovery V20) was illuminated through a fluorescence filter (ZEISS, filter set 38 HE, 470±20 nm, 0.24 mW/mm^2^). For whole-body illumination, light from an LED source (Optocode, LED-EXSA) was delivered to the entire body of each nematode. To use light of different wavelengths, we changed the LED head accordingly (red, EX-660; green, EX-530; blue, EX-450; UV, EX-365). When nematodes ceased forward movement and began backward movement within 5 seconds after light irradiation, it was considered as “light-avoidance behavior.” In a previous *C*. *elegans* study [7], light-avoidance behavior was defined as backward movement within 3 s of light irradiation. However, in this study, we defined it as within 5 s because the locomotion speed of *P*. *pacificus* is slower than that of *C*. *elegans*. In Fig 3C, because only *C*. *elegans* was assayed, backward movement within 3 s was defined as light-avoidance behavior. For each individual, the light avoidance assay was performed five times at 10 min intervals after each assay. A red filter (Kenko, 158371) was used to minimize the impact of white light from the lower part of the microscope. All assays were performed in a blinded manner.

The light intensity was measured using an optical power meter (HIOKI, 3664) with an optical sensor (HIOKI, 9742) divided by the illuminated area. Except for Fig 1, the intensity of blue light (470 nm) was 0.24 mW/mm^2^ (*P*. *pacificus*) or 1.83 mW/mm^2^ (*C*. *elegans*) for the light avoidance assay.

### Protein sequence collection

We used previously published datasets and BLASTP sequence searches to collect a reference dataset for photoreceptor protein families. Specifically, for the opsin and cryptochrome/photolyase families, we utilized the data collected by Gühmann et al, 2022 [44] and Deppisch et al, 2022 [45]. For the LITE-1 family, we performed BLASTP (https://parasite.wormbase.org/Multi/Tools/Blast) searches on all nematode species registered in WormBase ParaSite (Version: WBPS18, https://parasite.wormbase.org) [46] using the *C*. *elegans* LITE-1 (WBGene00001803) protein sequence as query. From this result, we obtained 88 sequences using BioMart (https://parasite.wormbase.org/biomart/martview/). Furthermore, we added the sequences of *Cel-*EGL-47 (WBGene00001211), *Cel-*GUR-3 (WBGene00001804), and *D*. *melanogaster* Gr28b (FBgn0045495, from FlyBase; https://flybase.org) [71] which are registered as paralogs or orthologs of *Cel-*LITE-1 in WormBase (Version: WS291, https://wormbase.org) [72,73] to the LITE-1 family (S1 Data). These three protein sequence files were used as inputs for subsequent analyses.

### Exploration of putative photoreceptor proteins

Protein domain searches were performed on the photoreceptor protein reference dataset and all proteins of *P*. *pacificus* based on the Pfam database (version 36.0) [74] using InterProScan (version 98.0, option: -dp -appl Pfam) [75]. Protein domains that were functionally important as photoreceptor proteins in the reference dataset were isolated and searched in all protein domain dataset of *P*. *pacificus*.

Orthology clustering with OrthoFinder (Version: 2.5.5, option: -S blast -M msa) [76] was performed on the three photoreceptor protein reference datasets and the *P*. *pacificus* proteome (El paco V3, http://pristionchus.org) [77] to obtain orthogroup data (S1 Table). Orthogroups from OrthoFinder and domain prediction data from InterProScan were integrated to search for photoreceptor protein candidates from all proteins of *P*. *pacificus*.

Given that BLASTP, as used in OrthoFinder, may not detect remotely homologous genes, we performed PSI- and DELTA-BLAST (BLAST+, Version: 2.15.0; option: -comp_based_stats 1) for searching photoreceptor protein families in the proteome of *P*. *pacificus* (S2 Table). PSI- and DELTA-BLAST searches were iterated three times with the threshold set at 1e-3.

To determine whether any traces of known photoreceptor proteins remained in the genome, we performed tBLASTn (BLAST+, Version: 2.15.0) (option: -evalue 1e-3) on the genome and transcriptome of *P*. *pacificus*, using each photoreceptor protein reference dataset as a query.

### Sequence alignment

The protein sequences used for sequence alignment were candidate protein sequences from a homology search using OrthoFinder/BLAST and protein domain prediction using InterProScan. Sequences were aligned with MAFFT (version 7.525) [78] using the "—auto" option, specifically employing the L-INS-i method. The aligned sequences were visualized in R (Version 4.3.2) using the ggmsa package (Version: 1.3.4) [79].

### Genetic screen for light-unresponsive mutants

*P*. *pacificus* PS312 was mutagenized with ethyl methanesulfonate (EMS), as described previously [80]. Two methods were used to screen the light-unresponsive mutants. In the first method, the F1 worms were individually transferred onto *E*. *coli* plates. When the F2 animals reached the adult stage, a light-avoidance assay was conducted once per individual, with 10 individuals per plate. F2 strains exhibiting light avoidance at a frequency of 40% or less were selected, and F2 worms were individually transferred to *E*. *coli* plates. After a few days, the F3 worms were again tested for light avoidance. The mutants with impaired locomotion were excluded. In the second method, plates containing P0 were left for several days and F2 or F3 individuals were transferred individually to *E*. *coli* plates. Subsequently, primary and secondary screening were conducted in the same manner as in the first method. We screened more than 20,000 mutagenized F2 strains.

### Whole genome sequence

Five 6 cm NGM plates containing many adult worms were prepared. After collecting and washing the nematodes with M9 buffer, genomic DNA was purified using the GenElute Mammalian Genomic DNA Miniprep Kit (Sigma, G1N10). Whole genome sequencing (WGS) was performed by BGI JAPAN. The WGS data were mapped based on the method described by Rödelsperger et al, 2020 [81] to identify mutation sites. Briefly, Illumina read data were aligned to the El Paco genome assembly [26,77] using the BWA Mem program [82]. The initial variant call was generated using the mpileup command in BCFtools [83].

### Genetic mapping

Recombinant lines used for genetic mapping were obtained by crossing light-unresponsive mutants (derived from PS312) with a male wild-type strain RSA076. Light-unresponsive F2 individuals were isolated using a light avoidance assay. After laying eggs, F2 individuals were lysed using a worm lysis buffer. To confirm the light insensitivity of the F2 individuals, a light avoidance assay was repeated on the F3 individuals. Primers were designed around marker sequences from Pristionchus.org (http://pristionchus.org), which contained insertions or deletions in PS312 and RSA076. PCR with Dream Taq Green PCR Master Mix (Thermo Fisher Scientific, K1081) was used to determine the genotype. The primers used for chromosome mapping are listed (S5 Table). Some primers were adapted from a previous study [84].

### CRISPR/Cas9 mutagenesis

To generate CRISPR knock-out mutants, we followed previously described co-injection marker methods [85–87]. The CRISPR target sequences were designed using CHOP-CHOP v3 (http://chopchop.cbu.uib.no/) [88]. All tracrRNAs, crRNAs, and Cas9 proteins were synthesized by Integrated DNA Technologies (IDT). We mixed 0.5 μl of the Cas9 protein (10 μg/μl), 0.95 μl of crRNA (100 μM), and 0.9 μl of tracrRNA (100 μM), and incubated the mixture at 37°C for 15 minutes. Using the co-CRISPR system, we combined each RNP complex containing the gRNA of *Ppa-prl-1* and a target gene. For the fluorescence marker method, we added *Ppa-egl-20p*::*turboRFP* or *Ppa-eft-3p*::*turboRFP* (50 ng/μl) to the RNP complex and diluted with nuclease-free water up to 20 μl. The injection mixtures were microinjected into the gonads of young adult worms. The injected worms (P0) were placed individually on NGM plates. Approximately 24–48 hours later, P0 worms were removed from the plate. After 3–4 days, the F1 worms were screened for the presence of a roller phenotype or fluorescent worms. For mutation screening, a heteroduplex mobility assay was performed using microchip electrophoresis on MultiNA (Shimazu, MCE-202) or the DNA gel separation improvement agent Loupe 4 K/20 (GelBio). Sanger sequencing by Eurofins Genomics was used to determine the genotype. The identified mutants were subsequently backcrossed with the original wild-type strain (PS312) for at least three generations to eliminate off-target effects. The target sequences of the gRNA and primers are listed in S5 Table.

### Generating transgenic lines

The promoter regions were amplified using KOD One PCR Master Mix (TOYOBO, KMM-101). The lengths of the promoter sequences were as follows: *Ppa-daf-11*: 794 bp; *Ppa-tax-2*: 2401 bp; *Ppa-tax-4*: 3001 bp*; Ppa-grk-2*: 3001 bp. The promoter for *Ppa-daf-11* was constructed as described in a previous study [62]. For *Ppa-tax-2*, we used a region predicted to be a promoter in a previous study [89]. For *Ppa-tax-4* and *Ppa-grk-2*, we obtained a sequence of 3001 bp sequence upstream from the start codon. These promotors were cloned into vector containing codon optimized GFP, TurboRFP or *tetanus toxin* and *Ppa-rpl-23* 3’UTR [86]. The plasmids and genomic DNA of PS312 were digested using *Hind*III (pMO56, pMO59, and pMO81) or *Pst*I (pMO74). These transgenes (3–5 ng/μl), *Ppa-egl-20p*::*RFP* or *Ppa-egl-20p*::*GFP* (50 ng/μl) as co-injection markers, and genomic DNA (60 ng/μl) were injected into the gonad of young adult worms. The transgenic animals were screened under a fluorescence microscope (Leica, M165 FC or ZEISS, Discovery V20).

### Dye-filing

To identify the cell type of the amphid neurons (Figs 6A–6D and S1A–S1D) and assess dye-filing defects (S2A–S2F Fig) in amphid neurons, we followed previously described staining methods using the lipophilic dye DiI Stain (Thermo Fisher Scientific, D3911) or Fast DiO Solid (Thermo Fisher Scientific, D3898) [28,39,40]. Well-fed J2 or J3 larvae (for cell identification) or adult (for dye-filing assay) were collected in M9 buffer and centrifuged at 1500 × g for 2 min. After discarding the supernatant, worms were incubated with 150 μl of M9 containing a 1:150 dilution of FastDiO or a 1:74 dilution of DiI for 1.5–3 h at 20°C. The nematodes were washed three times with 1 ml of M9 buffer and crawl freely on *E*. *coli* seeded NGM plates for more than 1 h. Worms were immobilized on 2% agarose pads containing 5 mM levamisole or 0.3% sodium azide and covered with a cover slip. Z-stack images were obtained using a confocal microscope (Zeiss, LSM900). The cell type of the amphid neurons was identified by analyzing the positional relationship between the stained cells and cells expressing the fluorescent protein. Reporter-positive cells were defined if the reporter fluorescence was observed in those cells in more than 80% of the individuals. To evaluate the dye-filing defect, maximum projections were generated using Fiji software [90]. A clear DiI signal in the cell bodies of amphid neurons was counted as a positive staining.

### Statistical analysis

The Prism software package GraphPad Software 9 was used for statistical analyses. Information about the statistical tests, p-values, and n numbers is provided in the respective figures and figure legends. All error bars show SEM.

## Supporting information

S1 Fig CNG channels are expressed in amphid neurons and *Ppa-grk-2* is expressed in various tissues. (A-D) Representative cell identifications of *Ppa-tax-2p*::*RFP* (A), *Ppa-tax-4p*::*RFP* (B), *Ppa-grk-2p*::*GFP* (C, D). (A) and (B) are maximum projection images. (C) and (D) are single focal plane images. FastDiO (shown in green in A and B) and DiI (shown in magenta) stained AM1, 2, 3, 4, 8, 9, and 11. *Ppa-egl-20p*::*GFP* or *RFP* was used as an injection marker and was expressed in AM5. Scale bars = 10 μm. (E) Representative fluorescence images of *Ppa-grk-2p*::*GFP* in whole body. Left is merged image of DIC and fluorescence, and right is fluorescence image. GFP was expressed in pharyngeal muscles, head neurons, body wall muscles, ventral nerve cord, and tail neurons. Scale bars = 10 μm.
(TIFF)

S2 Fig Cilia-related mutants showed normal light avoidance. (A-E) Dye-filling staining of amphid neurons in wild type (PS312, A), *Ppa-daf-19* (*tu1035*, B), *Ppa-osm-1* (*tu1129*, C), *Ppa-osm-12* (*tu1099*, D) and *Ppa-che-3* (*tu1416*, E) adults. Left are merged images of DIC and fluorescence, and right are fluorescence images. All images were generated by max projection. Amphid neurons were stained in wild type but not in cilia-related mutants. Scale bars = 10 μm. (F) Quantification of dye-filing staining in cilia-related mutants. (G) Blue light avoidance assay for cilia-related mutants. The mutants exhibited a normal percentage of light avoidance. One-way ANOVA Dunnett’s multiple comparison tests, compared with wild type. n.s. = not significant.
(TIFF)

S3 Fig Gene structures and CRISPR/Cas9 knock-out sites. Green boxes and orange arrowheads represent exons and target regions of the gRNA, respectively. Gene structures were based on El_Paco_annotation_V3 [77]. The illustrations were created by TBtools [91].
(TIFF)

S1 Movie Blue light avoidance in wild type *P*. *pacificus*. The wild-type strain PS312 moved to the bottom. After 5 s, blue light (470 nm) was applied to the head. At 8 s, the worm stopped moving and initiated backward movement.
(MP4)

S1 Table Orthogroup prediction data from OrthoFinder. (XLSX)

S2 Table PSI-/DELTA-BLAST and tBLASTn output results. (XLSX)

S3 Table List of *gur* genes in *P*. *pacificus* genome. (XLSX)

S4 Table All strains used in this study. (XLSX)

S5 Table All target sites and primers used in this study. (XLSX)

S6 Table Numerical data for all graphs. (XLSX)

S1 Data LITE-1 family protein sequences. (FASTA)
