# Supplementary figures and images for: cGMP-dependent pathway and a GPCR kinase are required for photoresponse in the nematode Pristionchus pacificus
Source: PLoS Genet. 2024 Nov 14;20(11):e1011320. doi: 10.1371/journal.pgen.1011320 (PMC11563456; doi:10.1371/journal.pgen.1011320)

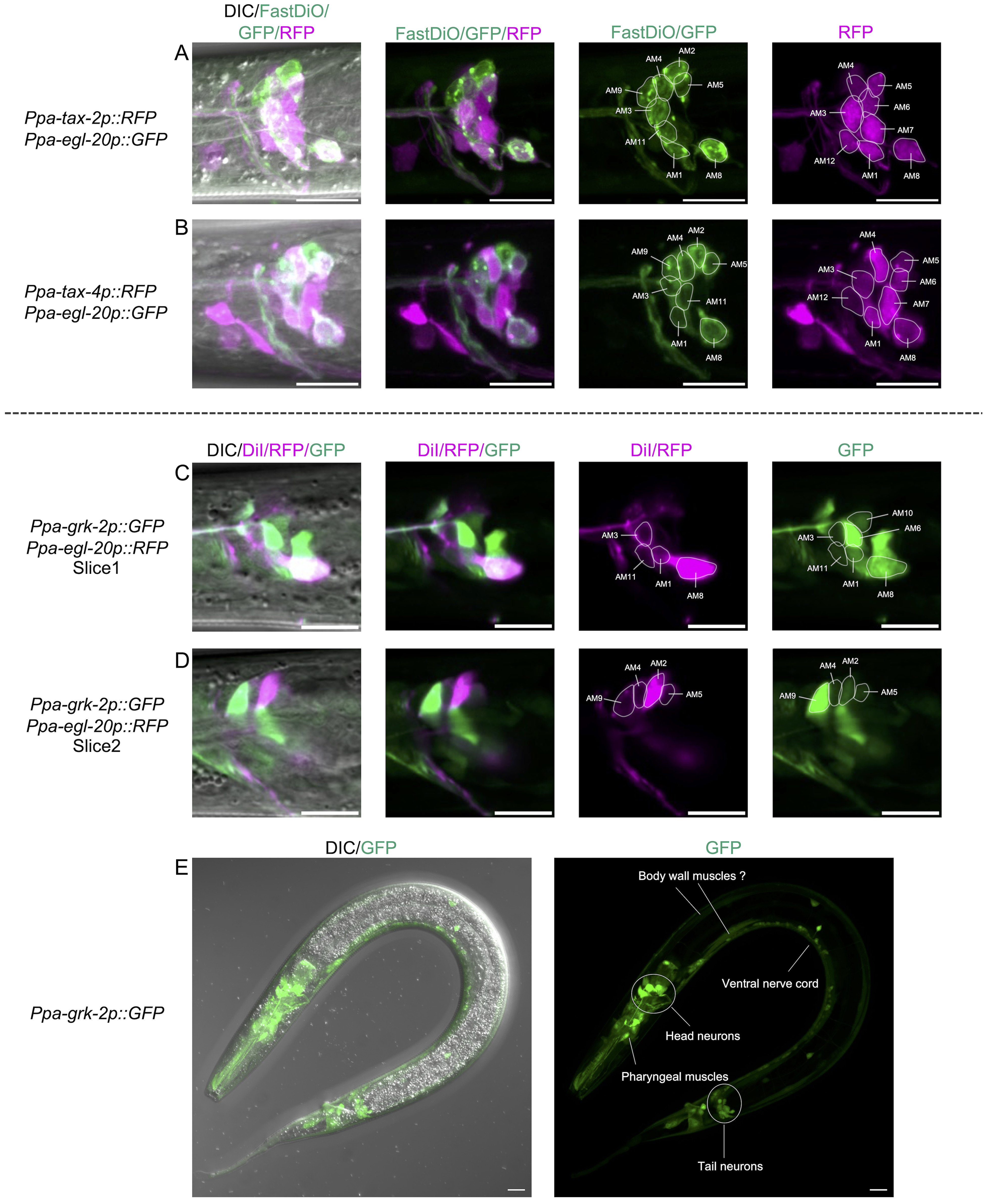

Supplement: S1 Fig — (A-D) Representative cell identifications of Ppa-tax-2p::RFP (A), Ppa-tax-4p::RFP (B), Ppa-grk-2p::GFP (C, D). (A) and (B) are maximum projection images. (C) and (D) are single focal plane images. FastDiO (shown in green in A and B) and DiI (shown in magenta) stained AM1, 2, 3, 4, 8, 9, and 11. Ppa-egl-20p::GFP or RFP was used as an injection marker and was expressed in AM5. Scale bars = 10 μm. (E) Representative fluorescence images of Ppa-grk-2p::GFP in whole body. Left is merged image of DIC and fluorescence, and right is fluorescence image. GFP was expressed in pharyngeal muscles, head neurons, body wall muscles, ventral nerve cord, and tail neurons. Scale bars = 10 μm. (TIFF) [file pgen.1011320.s001.tiff]

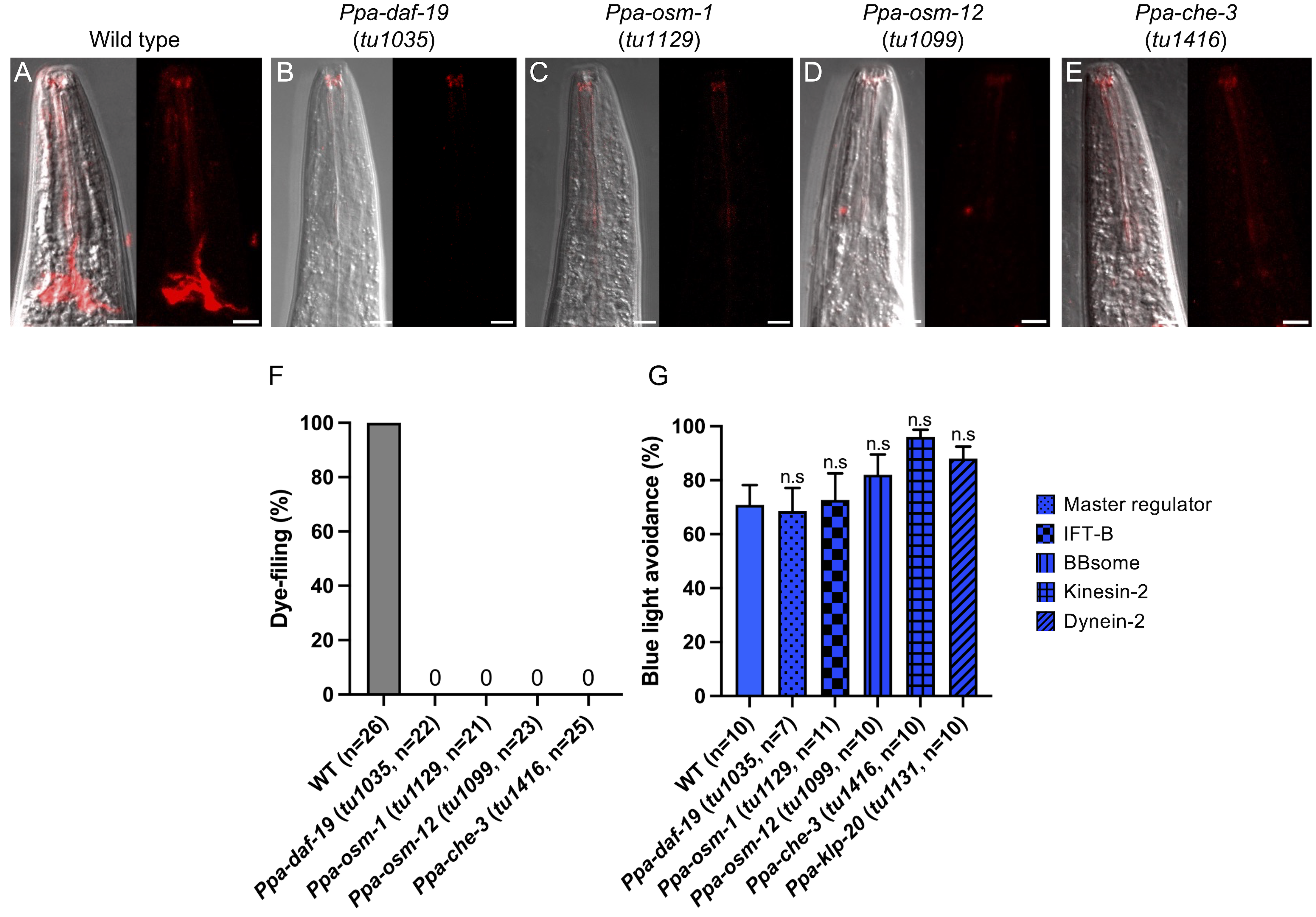

Supplement: S2 Fig — (A-E) Dye-filling staining of amphid neurons in wild type (PS312, A), Ppa-daf-19 (tu1035, B), Ppa-osm-1 (tu1129, C), Ppa-osm-12 (tu1099, D) and Ppa-che-3 (tu1416, E) adults. Left are merged images of DIC and fluorescence, and right are fluorescence images. All images were generated by max projection. Amphid neurons were stained in wild type but not in cilia-related mutants. Scale bars = 10 μm. (F) Quantification of dye-filing staining in cilia-related mutants. (G) Blue light avoidance assay for cilia-related mutants. The mutants exhibited a normal percentage of light avoidance. One-way ANOVA Dunnett’s multiple comparison tests, compared with wild type. n.s. = not significant. (TIFF) [file pgen.1011320.s002.tiff]

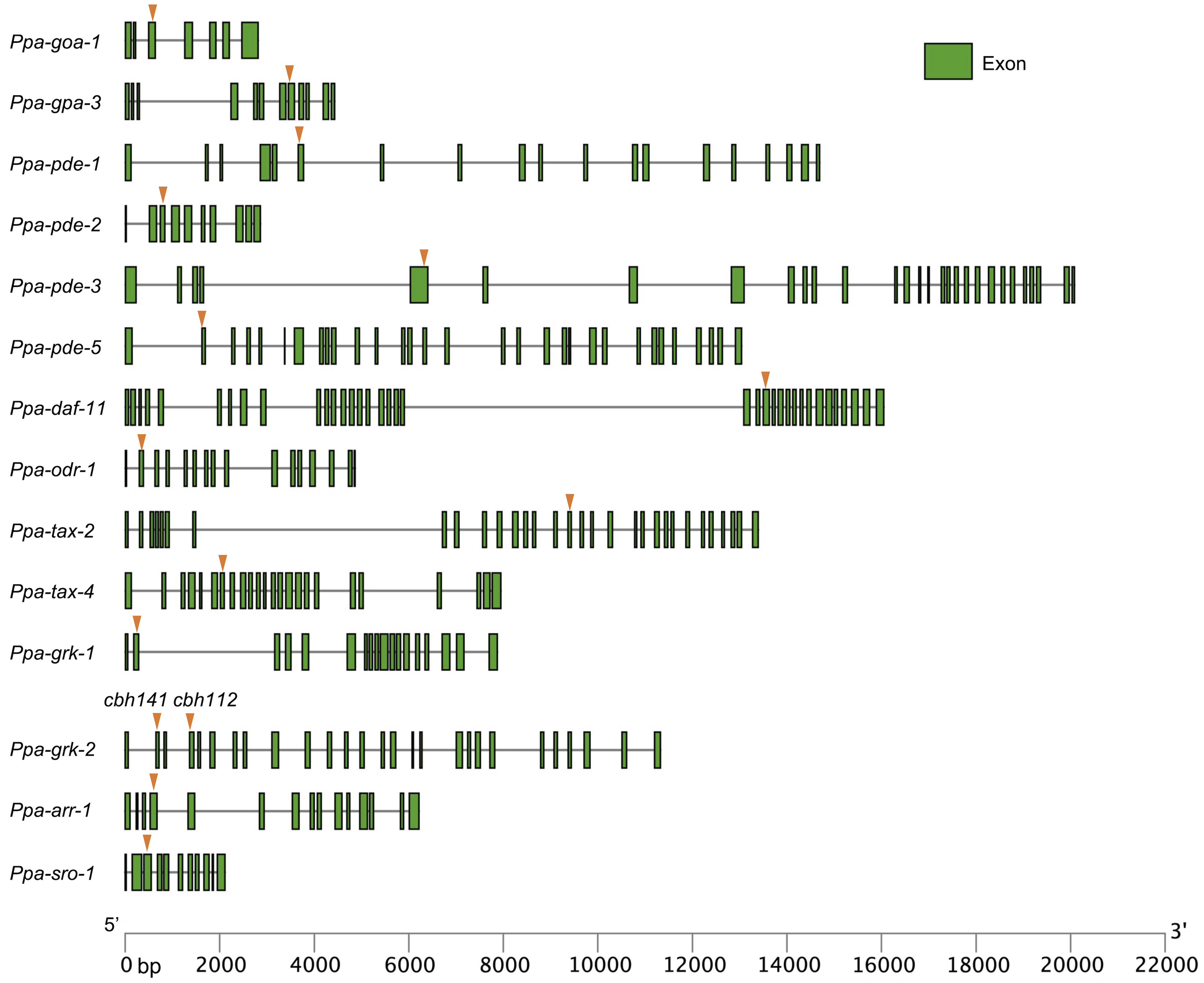

Supplement: S3 Fig — Green boxes and orange arrowheads represent exons and target regions of the gRNA, respectively. Gene structures were based on El_Paco_annotation_V3 [77]. The illustrations were created by TBtools [91]. (TIFF) [file pgen.1011320.s003.tiff]
